# Role of IFN-α in Rheumatoid Arthritis

**DOI:** 10.1007/s11926-023-01125-6

**Published:** 2023-12-05

**Authors:** Chung M. A. Lin, John D. Isaacs, Faye A. H. Cooles

**Affiliations:** 1 Translational and Clinical Research Institute, Newcastle University, Newcastle upon Tyne, UK; 2 Musculoskeletal Unit, Newcastle upon Tyne Hospitals NHS Foundation Trust, Newcastle upon Tyne, UK

**Keywords:** Rheumatoid arthritis, Early rheumatoid arthritis, Type 1 interferons, Interferon gene signature, Biomarkers

## Abstract

**Purpose of Review:**

Type 1 interferons (IFN-I) are of increasing interest across a wide range of autoimmune rheumatic diseases. Historically, research into their role in rheumatoid arthritis (RA) has been relatively neglected, but recent work continues to highlight a potential contribution to RA pathophysiology.

**Recent Findings:**

We emphasise the importance of disease stage when examining IFN-I in RA and provide an overview on how IFN-I may have a direct role on a variety of relevant cellular functions. We explore how clinical trajectory may be influenced by increased IFN-I signalling, and also, the limitations of scores composed of interferon response genes. Relevant environmental triggers and inheritable RA genetic risk relating to IFN-I signalling are explored with emphasis on intriguing data potentially linking IFN-I exposure, epigenetic changes, and disease relevant processes.

**Summary:**

Whilst these data cumulatively illustrate a likely role for IFN-I in RA, they also highlight the knowledge gaps, particularly in populations at risk for RA, and suggest directions for future research to both better understand IFN-I biology and inform targeted therapeutic strategies.

**Supplementary Information:**

The online version contains supplementary material available at 10.1007/s11926-023-01125-6.

## Introduction

**Fig. 1:**
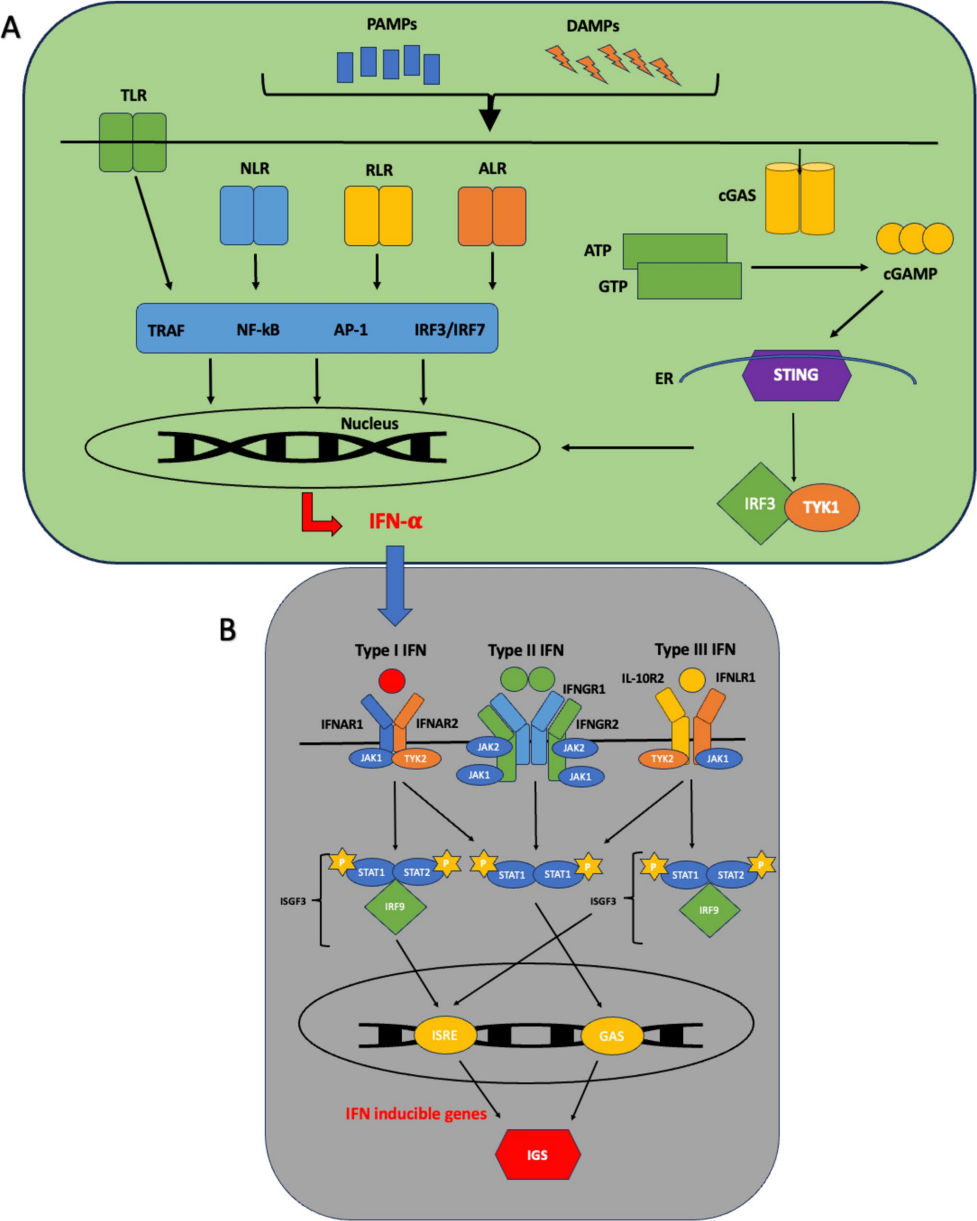
Schematic of interferon (IFN) triggers and downstream signalling pathways. **A** The production of IFN-I can occur following recognition of pathogen-associated molecular patterns (PAMPs), often associated with foreign bacteria or viruses, such as cytosolic DNA and double stranded RNA. These are detected by pattern recognition receptors (PRRs) which comprise of a large repertoire of germline-encoded receptors. These PRRs can be divided into subclasses including cell surface toll-like receptors (TLRs), cytosolic nod-like receptors (NLRs), retinoic acid inducible gene I receptors (RLRs), AIM2 like receptors (ALRs), and cGAS-STING pathway. Recognition of damage-associated molecular patterns (DAMPs) or PAMPS by PRRs results in transcription factor activation, such as TRAF (tumour necrosis factor receptor-associated factor), NF-kB nuclear factor kappa B, activating protein-1 (AP-1), and interferon regulatory factors (IRFs), STING (stimulator of interferon genes), and TBK1 (tank binding kinase 1), all involved in the transcription of IFN-I genes. **B** IFNs are categorised based on their receptor signalling, into IFN-I, IFN-II, and IFN-III. IFN-I signal via a heterodimeric receptor composed of two distinct multi-chain structures, IFN-α receptor 1 and 2 (IFNAR-1 and IFNAR-2) subunits. IFNAR associates with Janus Kinases (JAKs), with the former constitutively associated with JAK1 and the latter associated with tyrosine kinase 2 (TYK2). In response to ligand binding, these JAKs undergo activation and phosphorylate two latent transcription factors, signal transducers, and activators of transcription 1 and 2 (STAT1 and STAT2), resulting in their activation and subsequent heterodimer formation. This binds with IRF9 (IFN regulatory factor 9) or p48 to form a multi-component transcription complex called interferon-stimulated gene factor 3 (ISGF3). This complex translocates to the nucleus and binds to specific sites called IFN-stimulated response elements (ISREs), leading to the transcriptional induction of several IRGs ultimately responsible for IFN-I’s antiviral and immunomodulatory properties. The phosphorylated STAT proteins can alternatively form STAT1-STAT1 homodimers which bind gamma-activated sequences (GASs) to induce pro-inflammatory genes. As IFN-II can also signal via this alternative route (via their own heterodimeric receptor, composed of IFNGR1 and IFNGR2 subunits and associated with JAK1 and JAK 2 signalling), there can be a crossover between IFN-I and IFN-II signalling. Finally, IFN-III signals via its own heterodimeric receptor composed of IL-10R2 and IFNLR1 subunits, associated with the activation of TYK2 and JAK1, respectively. This can result in the formation and activation of STAT1-STAT2 heterodimers which associate with IRF9 to form ISGF3 complexes, with subsequent signalling as per IFN-I. AP-1, activating protein-1; DNA, deoxyribonucleic acid; ER, endoplasmic reticulum; NF-kB, nuclear factor kappa-light-chain-enhancer of activated B cells; NLR, nod-like receptor; P, phosphate; RLR, rig-I-like receptor; RNA, ribonucleic acid; TRAF, tumour necrosis factor receptor-associated factor.

Interferons (IFN) are a widely expressed family of cytokines. They are categorised, based on their receptor signalling, into types I, II, and III [1]. IFN-I signal via a heterodimeric receptor composed of two distinct multi-chain structures, IFN-α receptor 1 and 2 (IFNAR-1 and IFNAR-2). The former is constitutively associated with tyrosine kinase 2 (TYK2) and the latter associated with Janus Kinase 1 (JAK1) [2]. IFN-I are produced as part of the innate immune response to infection and possess potent antiviral effects [2]. Triggers of IFN-I production and subsequent downstream signalling have been recently reviewed in [3] and is summarised in Fig. 1. Similarly, IFN-II and IFN-III signal via their own unique heterodimeric receptors composed of IFN-γ receptors 1 and 2 (IFNGR-1 and IFNGR-2), IFNLR1 (IFN lambda receptor-1), and IL-10R2 (interleukin-10 receptor 2) subunits, respectively [4••]. Both of which subsequently lead to downstream signalling and potential induction of interferon inducible genes. In this review, we explore what role IFN-I, particularly IFN-α, may play in rheumatoid arthritis (RA) pathophysiology.

## The Interferon Gene Signature (IGS)

**Fig. 2:**
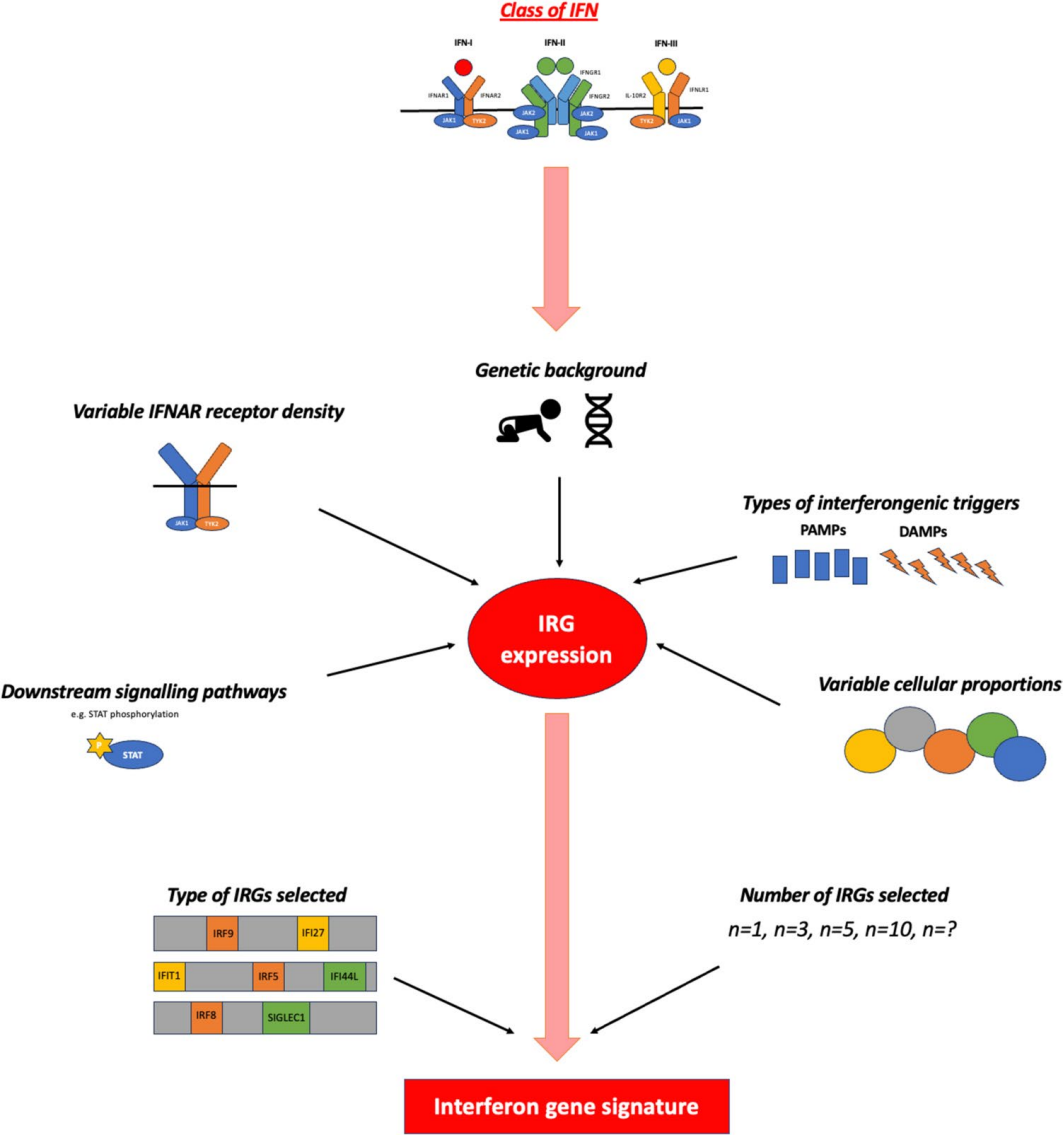
Figure highlighting factors that may influence interferon response gene (IRG) expression, as well as additional aspects that can influence the subsequent calculation of the interferon gene signature (IGS). Primarily, class of IFN will dictate IRG expression and thus the resulting IGS calculated, however, additional contributory factors, for example genetic background or IFNAR expression, are highlighted. DAMP, damage associated molecular patterns; IFNAR, IFN alpha receptor; IFNGR, IFN gamma receptor; IFNLR, IFN lambda receptor; IGS, interferon gene signature; IRG, interferon response gene; PAMP, pattern associated molecular pattern; STAT, signal transducer and activators of transcription

Measuring IFN-α protein *in vivo* has been historically challenging due to low circulating levels being frequently below the detection thresholds of standard assays. A solution was to infer IFN-I exposure, and hence levels, by measuring transcripts that reflect interferon stimulated or response genes (IRGs), and their cumulative expression was termed the interferon gene signature (IGS) (see Fig. 2). However, there are over 2000 IRGs and which IRGs are chosen to generate an IGS is lacking consensus across studies [5••]. Despite this, an IGS is widely reported in autoimmune rheumatic diseases, and there are mutual IRGs increased in RA and other rheumatic diseases [6]. Nevertheless, some propose an exclusive and highly diverse IRG transcriptional profile in RA peripheral whole blood [7] as well as in synovial biopsy samples [8], distinct from that found in SLE. However, IRG expression, and subsequently the calculated IGS, may vary between different cell types, suggesting that differences seen amongst related autoimmune diseases could be secondary to different immune cell proportions and signalling pathway activation [9]. Indeed, variation is seen in flow cytometry detected STAT class phosphorylation in CD4+ T cells, CD8+ T cells, B cells, and monocytes following IFN-I stimulation [10].

As IFN-I, IFN-II, or even IFN-III can induce IRGs (see Fig. 1), there has been a historical lack of clarity as to which IFN class was responsible for the IGS in RA. Indeed, it remains a controversial topic as IRG expression may be modulated by additional stimuli, such as TNF-α, with variable effects reported in monocytes vs T cells [11]. Nevertheless, in established RA, there was reportedly equal contribution of IFN-α and IFN-β to the whole blood IGS vs IFN-α exposure being dominant in SLE [9]. However, in a cohort of nearly 200 early drug naïve RA patients, circulating IFN-α protein and not IFN-β, IFN-II, or IFN-III nor any other circulating inflammatory cytokine uniquely correlated with the whole blood IGS [12••]. This work remains to be validated, and reported differences may reflect disease stages, but does implicate predominantly IFN-α with the IGS in early RA.

Despite these caveats regarding its calculation, the IGS remains a useful tool in dissecting the role of IFN-I in RA, as explored below.

## The IGS by Disease Phase

It is increasingly appreciated that disease processes in early RA are likely to be distinct from established RA. In early RA, a raised IGS (*MxA*, *OAS1*, *ISG15*, *IFI44L*, *IFI6*) was more prevalent compared with established RA, approximately 50% vs 20% of patients, respectively [13], and fell with the initiation of therapy [12••, 13]. Therapeutics may contribute to a reduced incidence in established RA as glucocorticoids, as well as disease modifying anti-rheumatoid drugs (DMARDs), can modify the IGS [14]. Notably, this increase in early RA persists even after accounting for potential confounders such as disease stage dependant variation in cell subset proportions [15].

Corroborating the raised IGS noted at disease onset, there is emerging data that IFNs may contribute to the transition from preclinical to sustained clinical disease. In ontology studies and network pathway analyses, the IGS distinguished DMARD-naïve early arthritis patients that developed a persistent inflammatory arthritis from those that had a self-limiting course [16]. In ACPA+ arthralgia populations, i.e. those who are at risk for developing RA, an IGS increases the chance of progression to synovitis, and its inclusion in outcome models improved its predictive capacity [17, 18]. Even in healthy asymptomatic CCP+ individuals, there was evidence of increased IFN-α signalling which mirrored what was seen in early RA cohorts, and this, with other parameters, was able to differentiate progressors with a median of 4.1 years before symptom onset, from controls [19, 20•]. In seropositive and seronegative RA, as well as in high-risk seropositive arthralgia patients, there was an overlap in circulating cytokine profiles with IFN-α, as well as IL-5, and TNF-α upregulated up to 50% in seropositive arthralgia and seropositive RA patients but not in seronegative RA [21] with an odds ratio (OR) of 21 for RA development in seropositive arthralgia patients [18, 21].

## Clinical Characteristics and the IGS

There has been conflicting evidence around the impact of an IGS/IFN-I signalling on autoantibody production in RA. In established RA, there is a significant correlation between the IGS and ACPA titres and anti-carbamylated protein (anti-CarP) antibodies as well as with genes linked to B cell differentiation and antibody production [22]. Conversely, others found no relation between the IGS and the presence and/or titres of ACPA and RF in established disease [23]. Similarly, a 2016 systematic analysis, involving patients with established RA, found that there was no difference seen in the IGS between ACPA negative and ACPA positive patients [24]. Conversely, rheumatoid factor (RF) demonstrated a positive association between either the IGS or circulating IFN-α levels in both established and early RA as well as across several autoimmune rheumatic diseases [12••, 13, 25]. These differences may reflect disease stage but may also reflect variability in the IRGs chosen to represent the IGS, with some using a combination of 19 IRGs [24] and others using only 6 (IFI27, IFI44L, IFIT1, ISG15, RSAD2 and SIGLEC1) [25] for example.

Multiple observational studies in established RA have found no association between the IGS and disease activity [13, 24]. This contrasts with early drug naïve RA where in a number of prospective observational studies, a higher IGS in early drug naïve RA, were associated with increased baseline disease activity as well as a poorer response to initial therapies [12, 13, 15] which was validated in additional cohorts for specific IGSs [26].

RA, including early disease, is a risk factor for cardiovascular disease (CVD). [27, 28]. In mouse and human *in vitro* models, IFN-α stimulation of macrophages resulted in significant metabolic rewiring with over 500 metabolic genes, including those related to key processes in the pathophysiology of CVD such as glycolysis, oxidative phosphorylation, fatty acid synthesis, and lipid metabolism [29]. In addition, endothelial progenitor cells (EPCs), involved in vasculogenesis and repair, have impaired function in RA, with IFN-α implicated in both *in vitro* and *in vivo* studies [30–32]. In murine lupus models, prolonged and enhanced IFN-I exposure significantly reduced EPC numbers, with acute exposure affecting only EPC differentiation but not the cellular number [30]. Finally, IFN-α may also influence CVD by promoting insulin resistance given, as early as the 1980s, IFN-α was shown to impair glucose tolerance and insulin sensitivity [33] with reversal of this effect in IFNAR-/- mouse models [34].

## IFN-I and Its Effects on Cellular Function

### B and T Cells

IFN-I can widely influence B cell activity which may contribute to RA pathophysiology, for example, by supporting B cell survival via increased monocyte B-lymphocyte stimulator (BLyS) production, by direct stimulation of B cells, and indirectly through T cell and Dendritic Cells (DCs) stimulation [35]. Prolonged B cell survival can lead to increased differentiation into memory and plasma cells, immunoglobulin isotype switching, and autoantibody formation [36, 37]. Furthermore, IFN-α modifies the plasma cell transcriptome towards a proinflammatory phenotype [38]. IFN-I regulates BCR signalling, specifically via IFN-αR, which in turn may promote pathways involved in antibody formation and germinal centre development in murine models [39]. IFN-I can also influence the differentiation of CD4+ T cells towards a Th1 response [40], fostering B cell activation and subsequent activity [41]. IFN-I also promotes CD8+ T cell survival and CD8+ cytotoxic T cell activity as well as prolonging the proliferation and expansion of CD8+ antigen specific T cells via inhibition of apoptosis [42].

### Dendritic Cells

Dendritic cells (DCs) upregulate HLA-DR, CD40, CD80, and CD86 expression upon IFN-I exposure [43]. DC maturation and enhanced antigen presentation, in the context of increased co-stimulatory molecules, can result in the induction of autoimmunity in predisposed individuals via self-antigen presentation to low affinity autoreactive T cells [44]. In SLE susceptible mice, IFN-I-treated DCs showed relative apoptosis resistance, this activated DC longevity potentially contributing to the development of autoimmunity [45]. Conversely, in early drug naive RA, there was no association between the IGS and circulating CD1c or pDC frequency, but there was an inverse association with CD141+ DC frequency [46]. This highlights the DC subset dependant complexity of IFN-I signalling *in vivo*.

### Monocytes

Classical and non-classical monocytes have been implicated in RA pathogenesis [47]. How the IGS affects monocyte function *in vivo* in RA remains to be fully examined but, when exposed to IFN-Is *in vitro*, monocytes upregulate TLR7 and IRF expression, resulting in increased responsiveness to subsequent immunostimulatory ligands [48]. IFN-I exposure also increases expression of CD40, CD80, and CD86 and HLA-DR, ultimately promoting differentiation into a monocyte-derived dendritic cell, or mo-DC, with high capacity for antigen presentation [43, 49]. Mo-DCs are also known to be increased in the RA synovial compartment and promote Th17 differentiation [50]. However, as with DCs, what happens *in vivo* may be subset dependant as highlighted by enhanced responsiveness to IFN-α in murine proinflammatory monocytes secondary to increased IFNAR expression when compared with anti-inflammatory monocytes [51].

### Neutrophils

Neutrophils are one of the first cell types to enter the RA joint and may play an important role in the development and progression of RA [52]. They are a major contributor to the whole blood IGS in RA, attributed to their uniquely upregulated IFNAR expression, a phenomenon not seen in either healthy controls or RA PBMCs [53•]. Indeed, next generation sequencing of isolated blood neutrophils has found significantly upregulated IRGs in RA neutrophils compared to healthy controls [54]. How this increased sensitivity to IFN-I influences neutrophil function is being explored, but, intriguingly, the pathogenic phenotype proposed for RA consists of delayed neutrophil apoptosis, increased ROS production and chemokine expression which, in part, can be recapitulated by IFN-I exposure *in vitro* [55•].

### Fibroblasts

Synovial fibroblasts are resident cells in the stroma of joints [56], and we recently demonstrated comparable IFN-α levels in serum and early RA synovial fluid [12••]. In RA, these fibroblasts have an activated phenotype, characterised by resistance to apoptosis, and increased proliferation and production of inflammatory mediators that promote immune cell differentiation and survival [57]. Histology and RNA sequencing of early RA synovial tissue demonstrated three distinct pathotypes: fibroblastic pauci-immune, macrophage-rich diffuse myeloid, and a lympho-myeloid pathotype [58••]. In the lympho-myeloid pathotype, seven out of the eight differentially expressed blood transcripts in synovial versus whole blood were IFN-I responses genes (IFI27, ISG15, IFI44L, OASL, USP18, RSAD2, LY6E) [58•]. In addition, a pathogenic subset of sub-lining fibroblasts (*THY1*^*+*^*HLA*^*−*^*DR*^*high*^) have increased IRG expression [59]. However, this may not directly be secondary to IFN-I as TNF-α induced signalling co-opts the mTOR pathway to shift fibroblast like synoviocytes towards an IFN response [60] which has been shown to be via secondary autocrine production of IFNβ and subsequent activation of the IRF1-IFNβ-IFNAR-JAK-STAT1 axis [61]. Nevertheless, the role of IFN-α on fibroblast function in RA remains an important research question.

**Fig. 3:**
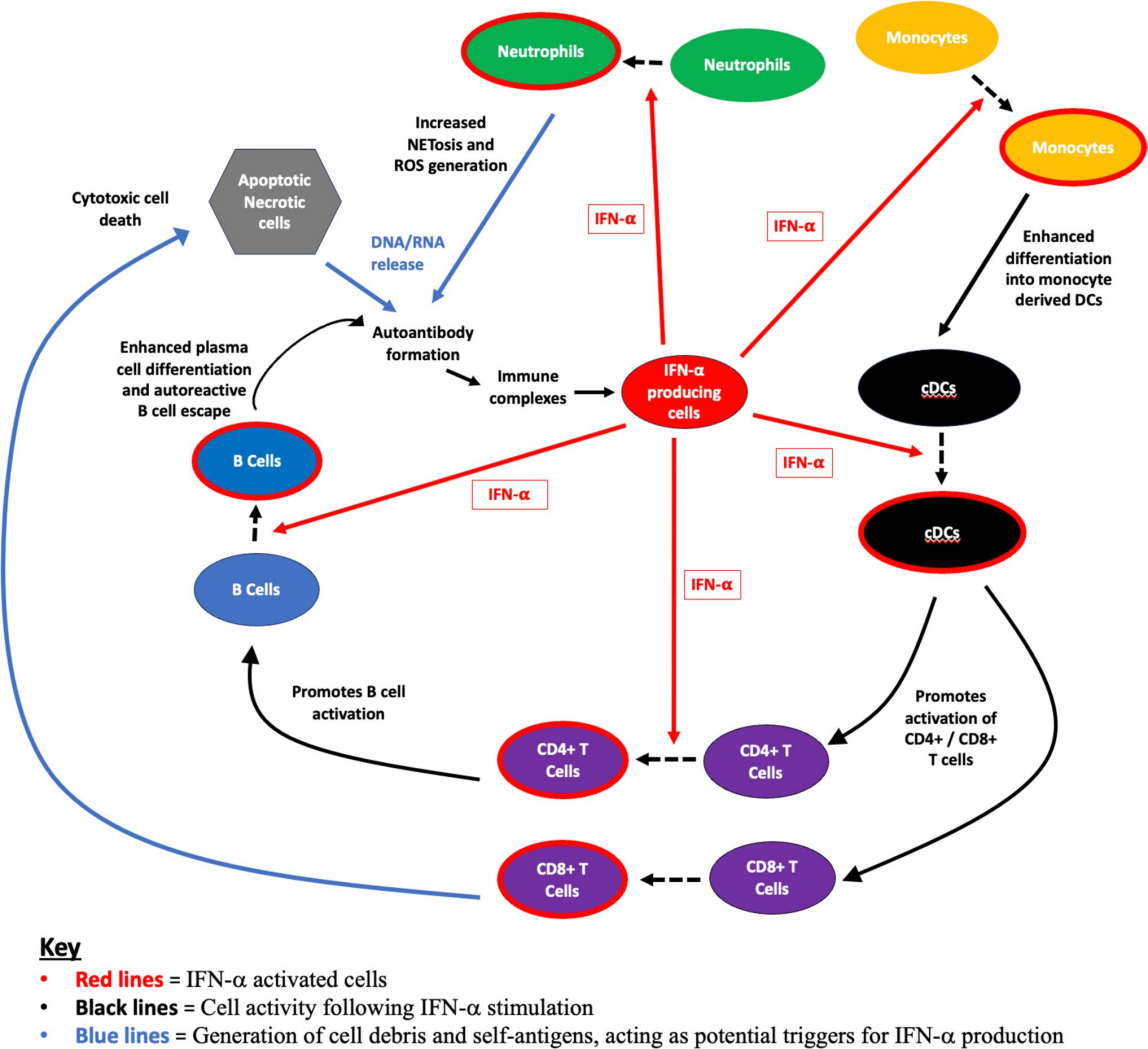
Schematic depicting interaction of cellular subsets in the presence of IFN-I. IFN-α influences the activity of surrounding innate and adaptive immune cells. It remains unknown what initially triggers the cascade of IFN production; however, it has been suggested that the generation of DNA/RNA via cell death pathways including apoptosis, necrosis, and NETosis (with subsequent ROS generation) plays a role. Exposure to these self-antigens increases the risk of developing autoantibodies, which form immune complexes that have potential to interact with IFN-producing cells to enhance further IFN-I production. Monocytes develop an inflammatory phenotype and activated cDCs promote activation of CD4+ and CD8+ T cell subsets. These T cells themselves upon exposure to IFN-I can further enhance B cell activation and mediation of cell death, respectively. cDCs, conventional dendritic cells; IFN-𝛂, interferon-𝛂; NET, neutrophil extracellular traps; ROS, reactive oxygen species

Cumulatively, these effects are likely to contribute to a highly activated and potentially autoimmune prone phenotype as summarised in Fig. 3.

## Source of IFN-I in RA

pDCs, particularly in their immature state, are the main IFN-I producing cell, however, whether they are the primary source of IFN-α in RA remains unclear. In SLE, there is an element of so-called pDC fatigue, where the ability of the pDC to produce IFN-I reduces and other cells take over production [62]. In early RA, circulating pDCs were not the primary source of *IFNA* transcript, with comparable expression in circulating lymphocytes. However, circulating pDC numbers were reduced with increased CCR7 expression inferring increased migration to the synovial compartment and target tissue [46]. Indeed, in established RA patients, the synovial compartment has increased numbers of pDCs with reduced numbers seen in peripheral blood. However, those that remained in the circulation were immature with inferred increased IFN-I producing capacity [63]. Nevertheless, RA synovial pDCs are potent producers of IFN-α [64] and, in mice, intraarticular transfer of IFN-I producing dendritic cells was sufficient to propagate a persistent inflammatory arthritis [65].

Conversely, after arthritogenic serum transfer in K/BxN serum-induced arthritis, collagen-induced arthritis, and human TNF transgene insertion, only pDC deficient mice showed exacerbations of symptoms and signs of inflammatory arthritis [66] and topical imiquimod, a TLR7 agonist, increased pDC recruitment and activity which subsequently improved arthritis [66]. Furthermore, transcriptomic analysis of circulating pDCs in early RA suggested enhanced tolerogenic function [46]. These discrepancies may arise due to the complexities of DC development [67] and cellular differences across species. Given their relative paucity *in vivo*, pDCs have been relatively neglected in RA research; however, better understanding of their complexity, particularly in relation to any location specific function, will help inform their role in RA and role in IFN-I production.

## Potential Triggers of IFN Production

What drives the observed increased IGS/IFN-α in RA remains unclear; however, triggers may include viral infections or microbial DNA or antigen fragments, with these elements repeatedly reported in the joints of RA patients [68–70]. Retroelements are non-protein encoding portions of DNA derived from ancient transposable elements, such as retroviruses, that have been historically incorporated into the genome. Their activity can trigger intracellular viral sensors and thus promote local IFN-I production [71]. In SLE and primary Sjogren’s syndrome, increased retrotransposon activity in disease relevant tissue associated with increased local IFN-α production [72•], and, in established RA synovium, there is also increased retroelement expression [73, 74]. Furthermore, in a subgroup of RA patients, a transcriptional profile was documented, reminiscent of a viral infection, which associated with both IFN-I signalling as well as increased ACPA titres [75, 76]. How these retroelements may influence IFN-I production in RA remains to be seen.

Cell-free nucleic acids have been extensively implicated in IFN-I generation in SLE [77] and monogenic interferonopathies [78]. Mouse models with DNA clearance defects develop autoantibody-mediated chronic polyarthritis, resembling human RA [79]. This corroborates RA human observational data, where evidence of raised levels of circulating cell -free DNA have been found in both peripheral blood [80, 81] and synovial fluid [82]. Although direct links with IFN-I were not made in these human RA studies, a similar mechanism to that described in SLE may be present*.*

Neutrophils, found in high numbers in RA synovium, can undergo NETosis, a unique form of cell death which has been proposed as a potential trigger for IFN-I production [83]. DNA from these NETs form complexes with antimicrobial peptides including LL37, secretory leukocyte protease inhibitor (SLPI), or with immunoglobulins to form immune complexes which facilitate pDC TLR7/9 signalling ultimately culminating in IFN-α production [84–86]. In RA, links between NETs and ACPA have been reported [87, 88] and known pathogenic cytokines in RA, such as TNF-α and IL-17A, as well as IFN-a itself [89], can also induce NETosis, potentially creating a vicious cycle of inflammation and disease activity [88].

Other potential triggers include lifestyle and environmental factors. An inverse correlation between physical activity and IFN-I signalling has been reported [90]. In addition, physical activity was associated with downregulation of TLR and IL-17R signalling and reduced inflammatory cytokines production, including IFN-I [90].

**Fig. 4:**
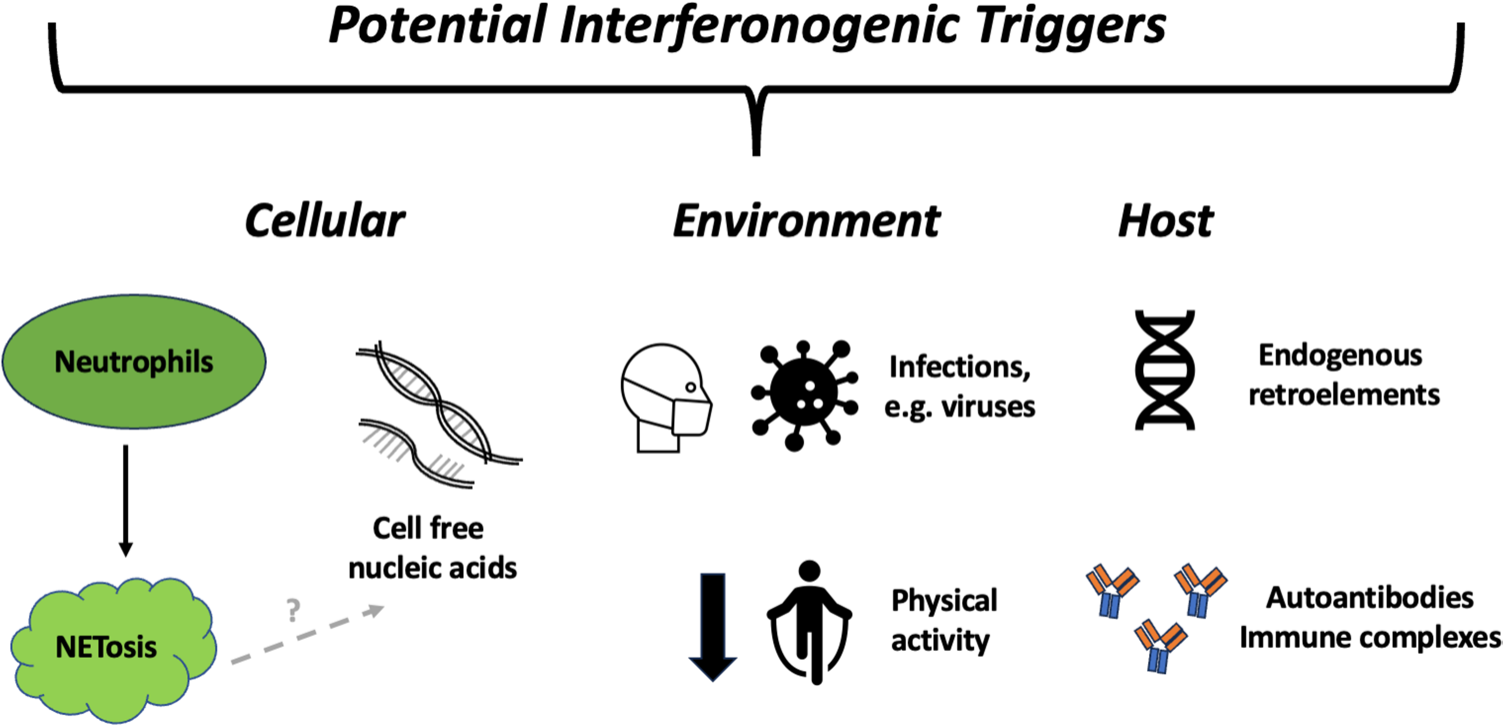
Figure depicting some potential triggers of IFN-α production. Here, potential triggers are split into three subtypes: (1) cellular comprising of neutrophils, (2) environmental including infections increasing IFN-α production via cellular death and debris and a reduction in physical activity reportedly linked to increased IFN-α levels, and (3) proposed non-cellular host triggers including endogenous retroelement activity and the development of autoantibodies or immune complexes resulting in increased IFN-α production

Potential triggers are summarised in Fig. 4; however, much of the above involves extrapolation from other diseases, such as SLE, and caveats exist including differences in IRG expression and genetic risk between these diseases [91]. Further work is needed to explore these pathways in RA specifically.

## Heritable Genetic Risk and IFN-I Signalling

As genome-wide association studies (GWAS), and relevant data sets, become more available, numerous single nucleotide polymorphisms (SNPs) have been identified as contributing to the genetic risk of RA. Interestingly, a number of these SNPs are in genes related to the IFN-I response pathway including DNA-sensing proteins, toll-like receptors, and JAK-STAT protein mediators. These are summarised in Table 1. However, the functional consequences of these polymorphisms in RA with regards to IFN-I production or signalling are yet to be elucidated. Nevertheless, an overlap of certain at-risk genes associated with increased IFN-I signalling in SLE has also been linked to RA, for example SNPs in IRF5, STAT4, and PTPN22 [92]. Some of these RA risk SNPs, such as IRF5 polymorphisms, associate with more severe or erosive disease [93, 94], which may corroborate the clinical refractory disease phenotype observed in IGS high early RA patients [12••]. Further work is needed to elucidate both the role of IFN-I on susceptible genetic backgrounds as well as the contribution of these SNPs to IFN-I production.

**Table 1:** Known single nucleotide polymorphisms (SNPs) associated with RA genetic risk and how their function may affect IFN-I biology

Gene with known RA risk variant	Role in IFN biology	Reference*
TNFAIP3 (A20)	• NF-κB and other A20-regulated signalling molecules can induce IFN-I	1
PADI4	• PADI4 knockouts resulted in reduced IFN-I responses	2
STAT4	• STAT4 promotes RIG-I signalling independent of its classical activation pathway and promotes IFN-β production in myeloid innate cells	3
CD40	• CD40 can enhance STING-mediated IFN-I responses	4
UBE2L3	• UBE2L3 shown to negatively regulate IFN-I expression	5
IFNAR1/IFNGR2	• Encodes signalling receptors for IFN-I and IFN-II	6
ETS1	• ETS-1 suggested in SLE patient studies to be associated with IFN-I and to negatively regulate ISG3 and ISRE binding sites	7,8
PVT1	• PVT1 negative feedback mediator for IFN-I signalling via STAT1 interaction and subsequent reduction of its phosphorylation. • IFN-α stimulation shown to upregulate PVT1 RNA expression	9,10
CDK6	• CKD6 regulates IFN-I signalling negative feedback loops	11
ETV7	• ETV7 negatively regulates IFN-I signalling	12
EOMES	• EOMES expression is driven by IFN-I signalling in CD8+ T cells which leads to regulation of memory-like CD8+ T cell homeostasis and function	13
TYK2	• TYK2 required for IFN-I induced activation of transcription factors STAT1-4 and downstream signalling	14
IRF8	• Regulates IFN-I production	15
Runx1	• RUNX1 upregulates IFNs and IRGs via IFN-I signalling	16
RCAN1	• RCAN1 protein stability negatively affected by IFN-α treatment via STAT2 activation	17
GATA3	• GATA3 overexpression promotes IFN-I expression • IFN- α/β treatment suppresses GATA3 expression	18,19
DDX6	• DDX6 regulates RIG-I mediated IFN-I signalling • DDX6 depletion leads to increased IRG expression	20,21
PRDM1	• Deletion results in impaired IFN-I production • Control IKKα and IRF7 activation via direct suppression of Irak3, a negative regulator of TLR signalling	23
IRF5	• IRF5 shown to a positive regulator of IFN-I signalling • Risk haplotype of IRF5 associated with SLE and with increased IFN-I production	24

*Separate reference list in supplementary file 1.

## IFN-I and Epigenetics

Epigenetic changes are modifications that regulate genome activity, independent of DNA sequence. This occurs via molecular factors and processes, such as DNA methylation of CPG sites or chromatin conformational changes, which subsequently modulate transcription. They are frequently triggered by environmental factors or exposure to inflammatory stimuli, such as cytokines. Methylation changes are noted early in RA progression and vary by cell subset [95••]. Furthermore, differential methylation has been implicated in initial response to methotrexate in early drug naïve RA patients as well as to certain biologics in established disease [95••, 96–98], and these processes are emerging as important modifiers of RA clinical progression and phenotype [99].

Analysis of B and CD4 T cells from early drug naïve RA patients demonstrated differentially methylated CPG sites at disease relevant genes, such as PARP9, STAT1, and EPSTI between IGS high and low patients. It also implicated altered transcription factor binding, cumulatively promoting increased lymphocyte activation, and a proliferative phenotype in the IGS high cohort [12••]. These data suggest that these changes may be IFN-α induced, and negatively influence clinical trajectory. In undifferentiated arthritis (UA) monocytes, methylation changes, which associated with disease progression and a poor prognosis, were partially recapitulated by monocyte exposure to IFN-α [100•]. Furthermore, IFN-α treatment causes methylation changes in monocytes similar to those seen in established RA, which *in vivo* were themselves associated with increased disease activity [101]. Intriguingly, in models of type 1 diabetes, where IFN-I plays a key part in disease initiation, exposure to IFN-α triggered increased TET2 expression. This prompted hypomethylation changes in genes controlling inflammatory and immune pathways, ultimately resulting in their increased expression and disease acceleration [102]. TET proteins are key players in demethylation and are also increased in early drug naïve RA circulating lymphocytes [103], however whether this is secondary to IFN-α is unknown.

It is important to acknowledge that CPG sites in IRGs themselves are frequently hypomethylated in autoimmune conditions, including RA [104, 105]. In twin studies of CD4 T cells, hypomethylation of IRGs *IFIT1*, *IRF7*, *MX1*, *OAS1*, *USP18*, *RSAD2,* and *IFI44L* has even been proposed as biomarkers of progression to RA [104]. This questions whether the IGS could be an artefact of altered gene expression secondary to hypomethylation caused by other circulating inflammatory cytokines, such as IL6, or due to increased IFN-α signalling itself. IFN-α protein is increased in early RA and uniquely correlates with the IGS [12••], so the reality is likely to involve both mechanisms.

Although less extensively investigated, IFN-I-associated chromatin conformational changes may also be relevant to RA pathophysiology. There is variation in chromatin accessibility in RA synovial fibroblasts which is likely influenced by the synovial environment [106], where IFN-α is known to be present [12••]. In early RA, chromatin conformation changes in *IFNAR2* were associated with poorer outcomes [107]. Furthermore, monocytes stimulated with IFN-α have increased trimethylated histone H3 Lys 4 (H3K4me3) which enhances transcription at promotors of genes that encode inflammatory mediators. In a more representative *in vivo* environment, incubation of monocytes with both IFN-α and TNF-α was associated with increased H3K4me3 that reduced monocyte tolerization to LPS and promoted an enhanced response to subsequent environmental challenges [108]. This intriguingly implicates IFN-I, and chromatin-mediated modifications, with the induction of inflammatory genes beyond canonical IRGs. Indeed, instances where prior exposure to IFN-α can influence cellular response to additional stimuli are increasingly being reported [102, 109–111] and remain a potential mechanism whereby IFN-I can influence disease development in RA.

## The IGS/IFN as a Therapeutic Target

Anifrolumab targets IFNAR1 and therefore blocks IFN-α and IFN-β signalling [112]. In a pilot trial, seven established RA patients, all with a high IGS (*IFI27, IFI44, IFI44L* and *RSAD2*), and active diseases were randomised to anifrolumab or placebo [113•]. The primary endpoint of an American College of Rheumatology (ACR) response of ≥ 20% after 24 weeks was achieved in patients receiving anifrolumab although only one patient in each arm completed the study despite a safety profile similar to that reported in SLE [114]. Reasons for early discontinuation in the treatment group included lack of efficacy, hypersensitivity reaction, and infection whilst the control group participants stopped due to insufficient therapeutic response [113•]. Larger trials are needed to assess the efficacy of this drug in RA.

Alternatively, JAK inhibitors (JAKi) suppress phosphorylation of STAT and thus affect downstream IFN signalling and reduce IRG expression [92]. Indeed, *in vitro* JAKi reduce IFN-I driven plasmablast differentiation [115], synovial BAFF expression, monocyte-derived DCs costimulatory molecule CD80/CD86 expression, and T cell differentiation into Th1 and Th17 cells [115, 116] [61]. The efficacy of JAKi in the treatment of established RA has been widely reported [117], although how the IGS impacts on its effect has not been comprehensively examined. However, analysis of baricitinib SLE trial data demonstrated that clinical effect was independent of IGS reduction [118].

Other inhibitors of downstream IFN-I signalling include a novel small molecule selective for JAK3/JAK1/TBK1 (tank-1 binding kinase), which, in mouse models, suppressed IFN-I production and osteoclast formation via TBK1 inhibition [119]. Autoantibody dependent collagen-induced arthritis mice models confirmed the clinical benefit of TBK1 inhibition [120, 121] and TBK1 deficient mice have reduced IRG and protein expression [122]. These findings are yet to be reproduced in human studies, but given the interest in cancer regarding TBK1 inhibition [123], this may provide a novel therapeutic approach.

## Conclusions

**Fig. 5:**
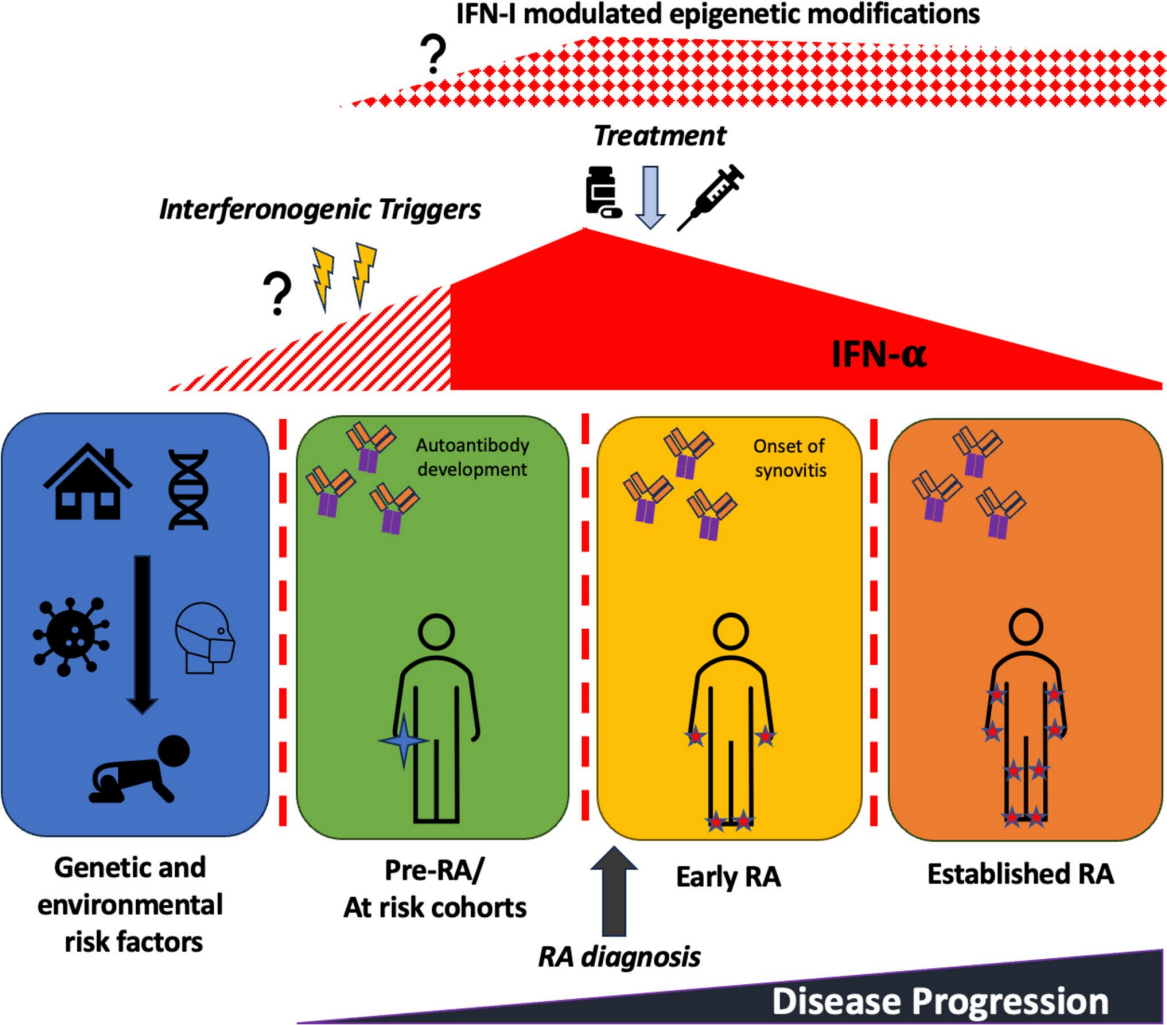
Schematic depicting associations between RA disease progression and IFN-𝛂 levels over time. There is increasing evidence that IFN-I is increased at RA disease onset and in at-risk cohorts. Proposed triggers include environmental influences including infection on the background of genetic risk; however, when these events may occur in relation to disease onset or initial immune dysfunction, with regards to autoantibody generation, is unclear. There is emerging evidence that this IFN-α exposure in early RA populations may cause potentially pathogenic epigenetic changes in key cellular subsets which could persist into established disease. IFN-𝛂, interferon-𝛂; RA, rheumatoid arthritis

There is growing evidence that IFN-α plays an important role in early RA pathophysiology and Fig. 5 summarises a working paradigm on IFN-α influencing RA progression. However, the triggers of IFN-α production and its timing in relation to early immune dysregulation or symptom onset remain unclear. Further work focusing on early disease or at-risk populations with a focus on genetic and epigenetic factors is likely to be informative. Despite mechanistic uncertainties, there is clear rationale to further test IFN-α targeting therapies in early RA, potentially using the IGS as a theragnostic biomarker, or to use the IGS as a biomarker for more intensive initial therapy. The heterogeneity and variety of IGSs remain challenging with regard to clinical utility, but recent progress in the international community on IGS stratification and uniform application of standardised measures of IFN-I signalling is encouraging [4••, 5••, 124, 125], and its use in this capacity may be on the horizon.

### Supplementary Information

ESM 1
