# Supplementary material for: Role of IFN-α in Rheumatoid Arthritis
Source: Curr Rheumatol Rep. 2023 Dec 5;26(2):37–52. doi: 10.1007/s11926-023-01125-6 (PMC10787895; doi:10.1007/s11926-023-01125-6)
Supplement: Supplementary file 1 — ESM 1 [file 11926_2023_1125_MOESM1_ESM.docx]

**Supplementary file 1.0**

1 Schwartz, D. M. *et al.* Type I interferon signature predicts response to JAK inhibition in haploinsufficiency of A20. *Ann Rheum Dis* **79**, 429-431, doi:10.1136/annrheumdis-2019-215918 (2020).

2 Liu, Y. *et al.* Peptidylarginine deiminases 2 and 4 modulate innate and adaptive immune responses in TLR-7-dependent lupus. *JCI Insight* **3**, doi:10.1172/jci.insight.124729 (2018).

3 Zhao, K. *et al.* Cytoplasmic STAT4 Promotes Antiviral Type I IFN Production by Blocking CHIP-Mediated Degradation of RIG-I. *J Immunol* **196**, 1209-1217, doi:10.4049/jimmunol.1501224 (2016).

4 Yao, X. *et al.* Increased CD40 Expression Enhances Early STING-Mediated Type I Interferon Response and Host Survival in a Rodent Malaria Model. *PLoS Pathog* **12**, e1005930, doi:10.1371/journal.ppat.1005930 (2016).

5 Feng, T. *et al.* Ubiquitin-conjugating enzyme UBE2J1 negatively modulates interferon pathway and promotes RNA virus infection. *Virol J* **15**, 132, doi:10.1186/s12985-018-1040-5 (2018).

6 Zanin, N., Viaris de Lesegno, C., Lamaze, C. & Blouin, C. M. Interferon Receptor Trafficking and Signaling: Journey to the Cross Roads. *Front Immunol* **11**, 615603, doi:10.3389/fimmu.2020.615603 (2020).

7 Suzuki, E. *et al.* The expression of Ets-1 and Fli-1 is associated with interferon-inducible genes in peripheral blood mononuclear cells from Japanese patients with systemic lupus erythematosus. *Medicine (Baltimore)* **101**, e31522, doi:10.1097/MD.0000000000031522 (2022).

8 Rutherford, M. N., Kumar, A., Haque, S. J., Ghysdael, J. & Williams, B. R. Specific binding of the ETS-domain protein to the interferon-stimulated response element. *J Interferon Cytokine Res* **17**, 1-10, doi:10.1089/jir.1997.17.1 (1997).

9 Ji, X., Meng, W., Liu, Z. & Mu, X. Emerging Roles of lncRNAs Regulating RNA-Mediated Type-I Interferon Signaling Pathway. *Front Immunol* **13**, 811122, doi:10.3389/fimmu.2022.811122 (2022).

10 Valadkhan, S. & Plasek, L. M. Long Non-Coding RNA-Mediated Regulation of the Interferon Response: A New Perspective on a Familiar Theme. *Pathog Immun* **3**, 126-148, doi:10.20411/pai.v3i1.252 (2018).

11 Klein, K. *et al.* T Cell-Intrinsic CDK6 Is Dispensable for Anti-Viral and Anti-Tumor Responses In Vivo. *Front Immunol* **12**, 650977, doi:10.3389/fimmu.2021.650977 (2021).

12 Froggatt, H. M., Harding, A. T., Chaparian, R. R. & Heaton, N. S. ETV7 limits antiviral gene expression and control of influenza viruses. *Sci Signal* **14**, doi:10.1126/scisignal.abe1194 (2021).

13 Martinet, V. *et al.* Type I interferons regulate eomesodermin expression and the development of unconventional memory CD8(+) T cells. *Nat Commun* **6**, 7089, doi:10.1038/ncomms8089 (2015).

14 Prchal-Murphy, M. *et al.* TYK2 kinase activity is required for functional type I interferon responses in vivo. *PLoS One* **7**, e39141, doi:10.1371/journal.pone.0039141 (2012).

15 Das, A., Chauhan, K. S., Kumar, H. & Tailor, P. Mutation in Irf8 Gene (Irf8(R294C) ) Impairs Type I IFN-Mediated Antiviral Immune Response by Murine pDCs. *Front Immunol* **12**, 758190, doi:10.3389/fimmu.2021.758190 (2021).

16 DeKelver, R. C. *et al.* RUNX1-ETO induces a type I interferon response which negatively effects t(8;21)-induced increased self-renewal and leukemia development. *Leuk Lymphoma* **55**, 884-891, doi:10.3109/10428194.2013.815351 (2014).

17 Lee, J. W. *et al.* The transcription factor STAT2 enhances proteasomal degradation of RCAN1 through the ubiquitin E3 ligase FBW7. *Biochem Biophys Res Commun* **420**, 404-410, doi:10.1016/j.bbrc.2012.03.007 (2012).

18 Li, J. *et al.* GATA3 Inhibits Viral Infection by Promoting MicroRNA-155 Expression. *J Virol* **96**, e0188821, doi:10.1128/jvi.01888-21 (2022).

19 Huber, J. P., Gonzales-van Horn, S. R., Roybal, K. T., Gill, M. A. & Farrar, J. D. IFN-alpha suppresses GATA3 transcription from a distal exon and promotes H3K27 trimethylation of the CNS-1 enhancer in human Th2 cells. *J Immunol* **192**, 5687-5694, doi:10.4049/jimmunol.1301908 (2014).

20 Zhang, R. *et al.* DEAD-Box Helicase DDX6 Facilitated RIG-I-Mediated Type-I Interferon Response to EV71 Infection. *Front Cell Infect Microbiol* **11**, 725392, doi:10.3389/fcimb.2021.725392 (2021).

21 Lumb, J. H. *et al.* DDX6 Represses Aberrant Activation of Interferon-Stimulated Genes. *Cell Rep* **20**, 819-831, doi:10.1016/j.celrep.2017.06.085 (2017).

22 Li, W. *et al.* Type I interferon-regulated gene expression and signaling in murine mixed glial cells lacking signal transducers and activators of transcription 1 or 2 or interferon regulatory factor 9. *J Biol Chem* **292**, 5845-5859, doi:10.1074/jbc.M116.756510 (2017).

23 Ko, Y. A. *et al.* Blimp-1-Mediated Pathway Promotes Type I IFN Production in Plasmacytoid Dendritic Cells by Targeting to Interleukin-1 Receptor-Associated Kinase M. *Front Immunol* **9**, 1828, doi:10.3389/fimmu.2018.01828 (2018).

24 Jefferies, C. A. Regulating IRFs in IFN Driven Disease. *Front Immunol* **10**, 325, doi:10.3389/fimmu.2019.00325 (2019).
